# Lung-Predominant Hyperinflammatory Syndrome in Therapy-Related Myelodysplastic Syndrome Responsive to Interleukin-1 Blockade

**DOI:** 10.3390/hematolrep18050068

**Published:** 2026-09-20

**Authors:** Quinn Smith, Anna Arar-Cauley

**Affiliations:** Department of Internal Medicine, University of Cincinnati Medical Center, Cincinnati, OH 45219, USA; araran@ucmail.uc.edu

**Keywords:** myelodysplastic syndromes, interleukin-1 receptor antagonist protein, inflammasomes, pneumonia, organizing, lung diseases

## Abstract

**Background/Objectives:** In myelodysplastic neoplasms, autoinflammatory syndromes result from dysregulated innate immunity and cause severe, organ-dominant inflammation. Interleukin-1 signaling through the NLRP3 inflammasome is implicated in the pathogenesis of myelodysplastic disease and related inflammatory complications, especially in the context of RAS-pathway activation. Reports describing therapy-related myelodysplastic syndrome with pulmonary autoinflammatory syndromes are rare, and the efficacy of interleukin-1 blockade in this setting has not been previously documented. **Results:** A man in his 60s with therapy-related myelodysplastic syndrome (del 5q) following rectal chemoradiation developed a recurrent, lung-predominant autoinflammatory syndrome refractory to corticosteroids and Janus kinase inhibition. Next-generation sequencing identified gain-of-function mutations in NRAS, PTPN11, SF3B1, and ASXL1. On two separate occasions, anakinra produced rapid clinical improvement with normalization of inflammatory biomarkers and radiographic resolution; on both occasions, discontinuation of anakinra led to prompt relapse within days, with ferritin rising to above 5500 ng/mL and C-reactive protein exceeding 300 mg/L. **Conclusions:** This case demonstrates that interleukin-1-mediated inflammation can drive pulmonary complications in therapy-related myelodysplastic syndrome. Targeted cytokine blockade with anakinra produced reproducible and measurable clinical improvement when conventional therapies were insufficient. The recurrence of symptoms following anakinra withdrawal, and rapid improvement upon re-administration, underscores the functional link between interleukin-1-driven inflammation and clinical outcomes. The RAS/MAPK mutational context and the observed on–off response to anakinra further support an interleukin-1-driven mechanism that merits further investigation.

## 1. Background

Inflammatory syndromes in myelodysplastic neoplasms are well recognized and include a wide spectrum of systemic autoimmune and autoinflammatory manifestations [1]. The underlying pathology is driven by increased bone marrow inflammation, aberrant immune activation, and dysregulated cytokine release. This includes interleukin-1 beta and interleukin-18 via NLRP3 inflammasome signaling [1,2,3,4]. Rare but severe conditions, such as VEXAS syndrome (Vacuoles, E1 enzyme, X-linked, Autoinflammatory, Somatic) and hemophagocytic lymphohistiocytosis, occur in fewer than 1% of patients with myelodysplastic neoplasms [2]. Pulmonary manifestations are less commonly reported but can produce refractory respiratory failure. This presentation is often difficult to distinguish from infection or drug-induced injury.

Mechanistic and translational studies have established a link between RAS-pathway activation and interleukin-1-dependent myeloid hyperinflammation. Clinical improvement has been observed with interleukin-1 receptor blockade [5]. In chronic myelomonocytic leukemia, patients with KRAS mutations have demonstrated benefit from the interleukin-1 receptor antagonist anakinra [6], providing a translational rationale for interleukin-1 inhibition in RAS-mutant myeloid autoinflammation. Pulmonary autoinflammatory syndromes in therapy-related myelodysplastic syndrome following chemoradiation represent a distinct and underreported clinical phenotype. The mechanistic overlap between RAS/MAPK activation, NLRP3 inflammasome signaling, and IL-1β-driven hyperinflammation, together with the emerging evidence for anakinra in RAS-mutant myeloid disorders, suggests a rationale for IL-1 blockade in this setting. However, formal documentation of this approach in therapy-related MDS with lung-predominant autoinflammatory syndrome has not been previously reported. We describe a case in which reproducible on–off anakinra response across two withdrawal-relapse cycles provides inferential evidence for IL-1 as the dominant pathologic driver.

## 2. Case Report

This case report is based on a retrospective review of an electronic medical record; infectious and rheumatologic evaluations are described in detail within the clinical narrative that follows. A man in his 60s was diagnosed with stage IIIB rectal adenocarcinoma (T3N1a, mismatch repair-intact) on routine colonoscopy. He underwent pelvic radiotherapy (50.4 Gy in 28 fractions) with concurrent fluorouracil (825 mg/m^2^/day infusion on radiation days), followed by adjuvant FOLFOX (folinic acid 400 mg/m^2^, fluorouracil 400 mg/m^2^ bolus then 2400 mg/m^2^ over 46 h, oxaliplatin 85 mg/m^2^ every 2 weeks). Pre-treatment blood counts were normal. Within a month of completing therapy, persistent pancytopenia led to a bone marrow biopsy, revealing hypocellularity, dysmegakaryopoiesis, and isolated 5q deletion (del 5q) with a blast count below 5%, consistent with therapy-related myelodysplastic syndrome with del(5q) per the WHO 2022 classification (IPSS-R intermediate risk). Cytogenetic analysis demonstrated an isolated del(5q) abnormality with no complex karyotype. Treatment began with luspatercept (1.0 mg/kg subcutaneously every 3 weeks) for anemia, followed by three cycles of lenalidomide (10 mg daily, days 1–21, every 28 days), the standard of care for 5q deletion-associated disease.

Within weeks, the patient presented to the emergency department with fever, fatigue, and dyspnea on exertion. Laboratory evaluation revealed markedly elevated inflammatory markers: ferritin greater than 6500 ng/mL and C-reactive protein of 163 mg/L. Chest computed tomography showed bronchial wall thickening and scattered mucus plugging. Repeat bone marrow biopsy showed rising blast counts. Because of concern for hemophagocytic lymphohistiocytosis or macrophage activation syndrome in the setting of a myeloid malignancy, dexamethasone (20 mg intravenously daily) and anakinra (100 mg subcutaneously daily) were started. The patient responded rapidly, with improvement in cell counts, ferritin, and C-reactive protein.

**Figure 1:**
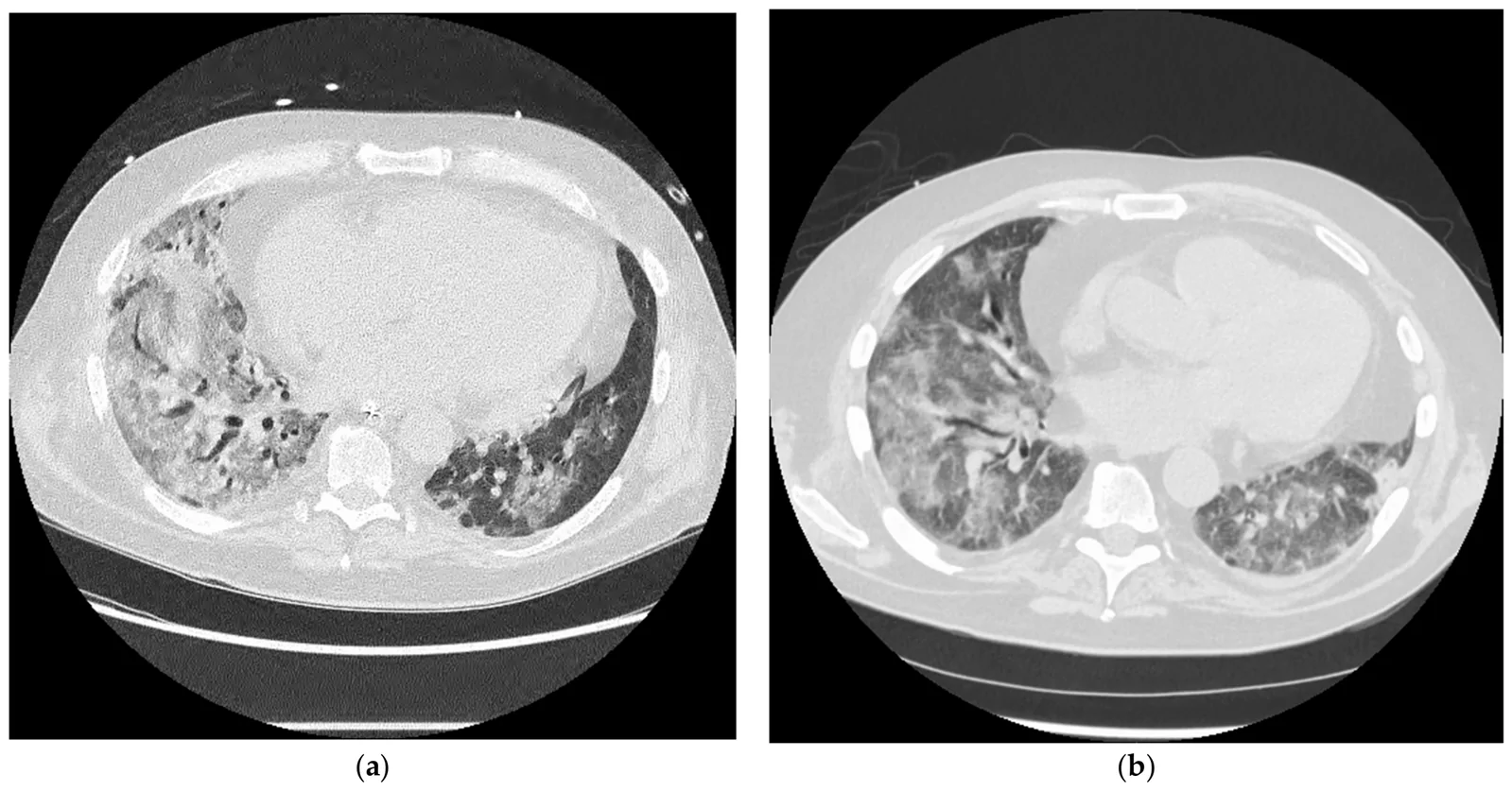
(**a**) Computed tomography of the chest without contrast demonstrating increased patchy consolidation in the right upper and middle lobes with adjacent ground-glass opacity, obtained during the second intensive care unit admission prior to anakinra initiation (authors’ original work). (**b**) Computed tomography of the chest without contrast demonstrating interval decrease in patchy ground-glass opacities throughout both lungs with interval increase in diffuse bronchiectasis, obtained following anakinra therapy. Compare with Figure 1a (authors’ original work).

Following a multidisciplinary discussion with the malignant hematology team, a treatment plan was established: azacitidine (75 mg/m^2^ subcutaneously on days 1–7 of each 28-day cycle) for two cycles, followed by a bone marrow biopsy and evaluation for allogeneic stem cell transplantation if blast counts remained below 5%. Shortly after initiating the first cycle of azacitidine, the patient developed worsening respiratory symptoms during a planned transfusion, requiring hospital admission and subsequent intensive care unit transfer. Computed tomography of the chest revealed ground-glass opacities predominantly in the right upper lobe (Figure 1a). Bronchoscopy with cryobiopsy was negative for infection but revealed clotting throughout the tracheobronchial tree, consistent with diffuse alveolar hemorrhage, with biopsy revealing an acute fibrinous-organizing pneumonia-like pattern with necrotizing inflammation [4,5]. The differential diagnosis for this presentation included infection, inflammation, or drug-induced lung injury. Symptoms improved with methylprednisolone (1 mg/kg intravenously daily with taper), and the patient was discharged in stable condition.

Shortly before his second cycle of azacitidine, the patient was readmitted with sacral cellulitis concerning for an abscess, requiring intravenous antibiotics. His hospital course was complicated by hypotension and progressive respiratory distress necessitating intubation and intensive care unit transfer. Repeat ferritin was 6683 ng/mL, and C-reactive protein was 225.9 mg/L. Given his prior response, anakinra was restarted at 200 mg subcutaneously daily, subsequently escalated to 200 mg twice daily, alongside high-dose intravenous methylprednisolone (2 mg/kg daily). Repeat bronchoscopy demonstrated hyperemic mucosa without diffuse alveolar hemorrhage. He was successfully extubated on day 6. Due to the absence of a diagnosis formally supporting anakinra use (e.g., hemophagocytic lymphohistiocytosis or a defined rheumatologic etiology), plans were made to wean both anakinra and corticosteroids after discharge. Repeat bone marrow biopsy showed an interval increase in blast counts. Next-generation sequencing (Day 37 of the clinical course) identified four somatic mutations: SF3B1 p.K700E (missense gain-of-function, VAF 15.9%), NRAS p.G13D (missense gain-of-function, VAF 4.5%), ASXL1 p.G607fs (frameshift loss-of-function, VAF 4.0%), and two concurrent PTPN11 mutations, p.D61N (VAF 24.0%) and p.D61V (VAF 12.9%), both gain-of-function variants at the same codon affecting the SHP2 phosphatase domain. Repeat sequencing performed approximately two months later demonstrated clonal expansion of NRAS p.G13D (VAF 4.5% to 13.2%) and ASXL1 p.G607fs (VAF 4.0% to 12.7%) despite cytotoxic therapy, while PTPN11 p.D61V was no longer detected, suggesting possible selective pressure or subclonal sampling variability. Treatment with decitabine (20 mg/m^2^ intravenously on days 1–5) and venetoclax (a BCL-2 inhibitor, 400 mg orally daily on days 1–28) was initiated to control the underlying disease process driving inflammation. During the following weeks, oxygen requirements improved markedly, becoming necessary only during sleep. Ferritin improved to 2221 ng/mL, and C-reactive protein decreased to less than 1 mg/L. Repeat computed tomography of the chest showed interval improvement in ground-glass opacification (Figure 1b). The patient was transitioned to ruxolitinib (10 mg orally twice daily) and prednisone (0.5 mg/kg daily with taper) and discharged to inpatient rehabilitation, with plans for a further cycle of decitabine and venetoclax and possible allogeneic stem cell transplantation.

Within one day of discharge, the patient returned with hypotension and overnight oxygen saturation in the 70s. Ferritin had risen to 5528 ng/mL and C-reactive protein to 300 mg/L. This deterioration represented the second documented anakinra withdrawal-relapse cycle: in cycle 1, anakinra was discontinued after the second ICU admission with initial improvement, but relapse ensued prompting rehospitalization; in cycle 2, anakinra was discontinued at discharge, and relapse occurred within 24 h, requiring a third ICU-level admission. Anakinra was restarted at 200 mg subcutaneously twice daily alongside high-dose intravenous corticosteroids. Intensive care was again needed due to increasing oxygen needs at rest.

**Figure 2:**
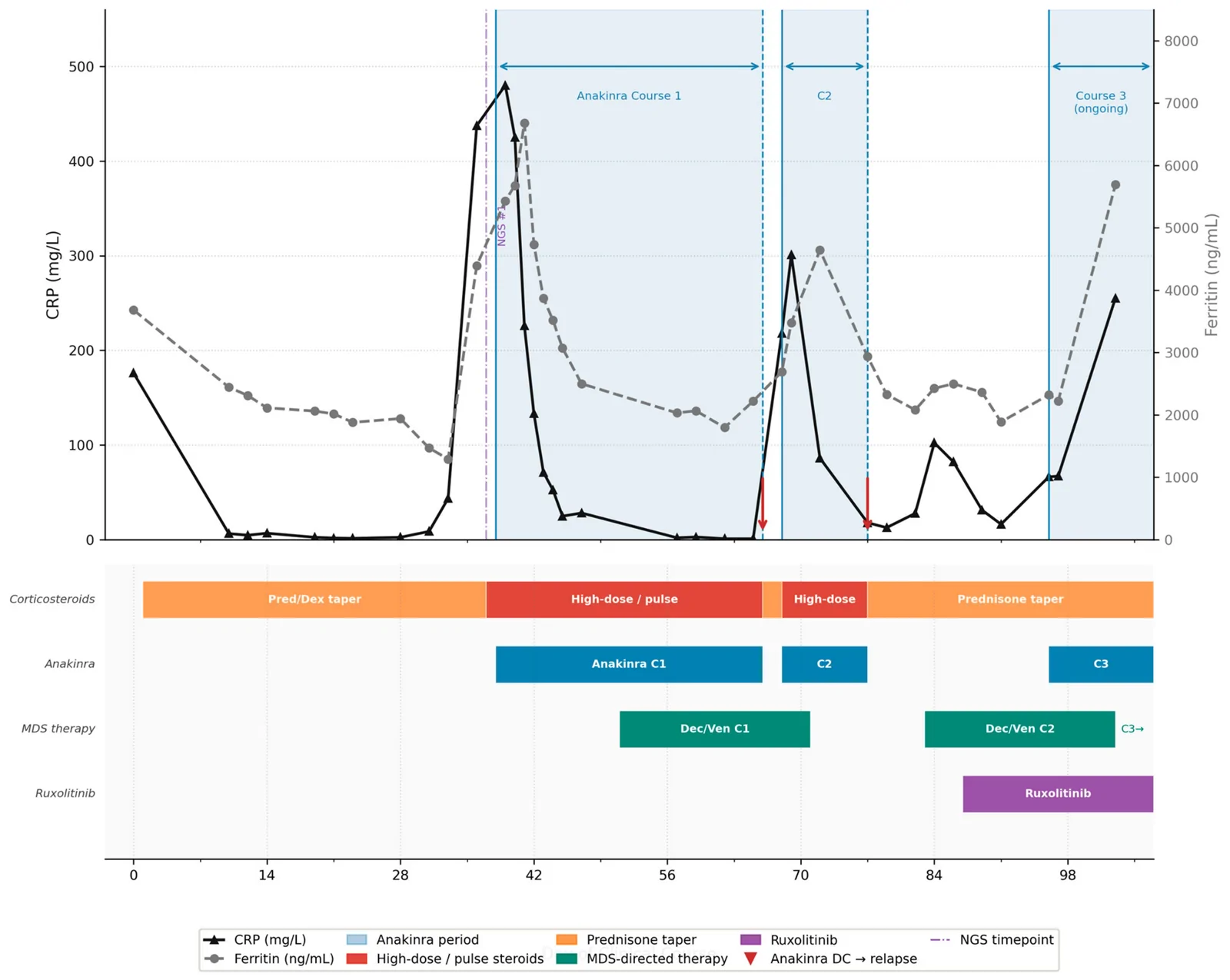
Serial inflammatory biomarkers and treatment timeline across 103 days of clinical course. Upper panel: C-reactive protein (CRP, left axis, mg/L; filled triangles) and ferritin (right axis, ng/mL; filled circles). Blue-shaded regions indicate anakinra administration periods. Course 1 (Days 38–66): anakinra initiated with marked reduction in both CRP and ferritin; discontinuation on Day 66 prompted rapid relapse (Cycle 1 withdrawal-relapse, indicated by red downward arrow). Course 2 (Days 68–77): anakinra re-initiated following relapse, then briefly discontinued; prompt relapse recurred within days (Cycle 2 withdrawal-relapse, red arrow). Course 3 (Day 96 onward): anakinra restarted with ongoing clinical response. Purple dash-dot line indicates timing of next-generation sequencing bone marrow evaluation (NGS #1, Day 37). Lower panel: concurrent treatment timeline showing corticosteroid phases (red = high-dose/pulse; orange = prednisone taper), anakinra courses, MDS-directed therapy, and ruxolitinib. DC = discontinuation; CRP = C-reactive protein; Dec/Ven = decitabine/venetoclax; NGS = next-generation sequencing (authors’ original work).

Data collected during multiple hospitalizations repeatedly pointed towards a waxing and waning inflammatory state (Table 1). Computed tomography of the chest demonstrated fluctuating multifocal ground-glass opacities and consolidations over three months (Figure 1a,b). Serial inflammatory biomarkers showed nadirs temporally associated with anakinra administration: C-reactive protein decreased from 225.9 to less than 1 mg/L, and ferritin from 6683 to 2221 ng/mL (Figure 2). Extensive infectious evaluation, including bacterial, viral, fungal, and Pneumocystis jirovecii assays, was repeatedly negative across multiple admissions. Three negative beta-D-glucan (Fungitell) assays, negative bronchoalveolar lavage fungal and Pneumocystis jirovecii testing, and non-angioinvasive imaging collectively argued against invasive fungal pneumonia, despite a single low-positive galactomannan result that was not corroborated on repeat testing. Rheumatologic evaluation did not identify an alternative autoimmune diagnosis.

## 3. Discussion

We report a man in his 60s with therapy-related myelodysplastic syndrome following rectal chemoradiation who developed a lung-predominant autoinflammatory syndrome that was reproducibly responsive to interleukin-1 blockade. Autoimmune and autoinflammatory manifestations are recognized complications of myelodysplastic neoplasms, with underlying pathology linked to chronic innate immune activation, bone marrow inflammation, and dysregulated cytokine release [1,2,3,7]. NLRP3 inflammasome signaling, with downstream interleukin-1β and interleukin-18 release, underlies both ineffective hematopoiesis and inflammatory complications in myelodysplastic syndrome [1,2,4].

Mechanistic data further link RAS-pathway activation to interleukin-1-dependent myeloid hyperinflammation. Oncogenic KRAS drives RAC1-ROS-mediated NLRP3 inflammasome activation in myeloid progenitors, resulting in constitutive interleukin-1β and interleukin-18 release [5]. The NRAS p.G13D variant identified in this patient activates the same RAS/MAPK effector pathway and has been associated with NLRP3-dependent hyperinflammation in myeloid disease. The two concurrent PTPN11 gain-of-function mutations (p.D61N, p.D61V) affect the SHP2 phosphatase domain and potentiate RAS-MAPK signaling, amplifying cytokine hypersensitivity and providing an additional mechanistic basis for interleukin-1 pathway overactivation [5]. Of note, serial sequencing demonstrated clonal expansion of NRAS p.G13D (VAF 4.5% to 13.2%) and ASXL1 p.G607fs despite cytotoxic therapy, suggesting that the mutational drivers of inflammation persisted and expanded during the clinical course, consistent with the ongoing inflammatory relapses observed after anakinra discontinuation. Cases in chronic myelomonocytic leukemia with KRAS mutations have demonstrated clinical benefit from anakinra, providing direct translational support for interleukin-1 receptor antagonism in RAS-linked myeloid autoinflammation [6].

Despite intensive corticosteroid therapy and Janus kinase inhibition with ruxolitinib, lung-predominant inflammation persisted in our patient. The reproducible on–off–on response to anakinra with objective improvement in oxygenation, computed tomography findings, and inflammatory biomarkers during each course, and rapid relapse within days of each discontinuation, provides strong inferential support for interleukin-1 as a dominant pathologic driver in this case (Figure 2) [6]. Two complete withdrawal-relapse cycles, each culminating in ICU-level respiratory failure and each reversed by anakinra re-initiation, are difficult to attribute to alternative mechanisms such as disease progression or infection alone, though concurrent corticosteroids, cytotoxic chemotherapy, and immunosuppression represent important confounders that preclude definitive mechanistic conclusions from a single case. This dosing strategy is consistent with published precedents in other hyperinflammatory conditions, including acute respiratory distress syndrome associated with severe SARS-CoV-2 infection, adult macrophage activation syndrome, and hemophagocytic lymphohistiocytosis, where interleukin-1-driven biology is suspected [7].

Hypomethylating agents remain the standard of care in myelodysplastic syndrome and have demonstrated evidence for controlling associated inflammatory disease [1,8,9]. Azacitidine was used as first-line therapy but was discontinued due to worsening inflammation and respiratory compromise. Decitabine combined with venetoclax was subsequently initiated, given disease progression on repeat bone marrow biopsy. It is worth noting that infectious etiologies remained a constant diagnostic concern throughout treatment, given the patient’s profound immune compromise. Acute fibrinous-organizing pneumonia is a recognized organizing pneumonia-spectrum pattern with variable steroid responsiveness; in hematologic settings, courses range from steroid-responsive to steroid-refractory, reinforcing an inflammatory rather than infectious adjudication [4,5]. Concomitant corticosteroid use and cytotoxic chemotherapy are confounding variables in the treatment course, and limited ruxolitinib exposure limits the ability to definitively assess Janus kinase inhibition as an alternative or adjunct therapy.

Several practical lessons emerge from this case. First, interleukin-1 blockade should be considered early in patients with myelodysplastic syndrome-associated hyperinflammatory syndromes that are refractory to corticosteroids, particularly in the presence of RAS-pathway or PTPN11 mutations. Second, anakinra should not be discontinued without a plan to transition to a steroid-sparing agent or address the underlying myeloid disease, given the risk of rapid relapse demonstrated here. Third, the absence of a formal diagnosis of hemophagocytic lymphohistiocytosis or a defined rheumatologic condition should not preclude empirical interleukin-1 blockade when the clinical phenotype and mutational context are consistent with interleukin-1-driven pathobiology.

Collectively, the clinical course, objective laboratory and radiographic findings, and the reproducible temporal association between anakinra administration and clinical improvement are consistent with a diagnosis of pulmonary hyperinflammatory syndrome in therapy-related myelodysplastic syndrome. This condition may be highly responsive to interleukin-1 inhibition, though controlled prospective data are needed to confirm this observation. The present case extends the observed benefit of interleukin-1 blockade in chronic myelomonocytic leukemia and RAS-mutant myeloid disorders to a therapy-related myelodysplastic syndrome pulmonary phenotype [1,2].

## 4. Conclusions

Therapy-related myelodysplastic syndrome may present with severe, lung-predominant autoinflammatory syndromes that are refractory to corticosteroids and Janus kinase inhibition. The RAS/MAPK mutational context (NRAS, PTPN11) and the reproducible on–off–on clinical response to anakinra observed in this case are consistent with an interleukin-1-driven pathology. Targeted cytokine blockade with anakinra resulted in objective improvements in oxygenation, inflammatory biomarkers, and radiographic findings, with rapid relapse upon discontinuation on two occasions, further supporting a potential mechanistic link. These findings support genotype-informed evaluation of interleukin-1 blockade in pulmonary autoinflammatory syndromes associated with myelodysplastic neoplasms and warrant further investigation in prospective studies.

## Figures and Tables

**Table 1:** Clinical timeline of autoinflammatory flares and treatment response.

Flare	Approx. Day	Symptoms/O_2_ Support	Peak CRP (mg/L)	Peak Ferritin (ng/mL)	CT Findings	Anakinra Dose	Corticosteroid	Response
Flare 1	Day 1–10	Fever, dyspnoea; supplemental O_2_ via nasal cannula	163	>6500	Bronchial wall thickening; mucus plugging	100 mg SC daily	Dexamethasone 20 mg IV then prednisone taper	Rapid improvement; discharged
Flare 2 (Cycle 1 relapse)	Day 11–38	Hypotension; progressive respiratory failure; intubation (PaO_2_/FiO_2_ ≈ 240)	480 (peak)	>7000	Ground-glass opacities right upper/middle lobe; AFOP pattern on cryobiopsy (Figure 1a)	200 mg SC daily → 200 mg SC twice daily	Methylprednisolone pulse dosing then taper	Extubated Day 6; anakinra weaned at discharge (Cycle 1 DC, Day 66)
Flare 3 (Cycle 2 relapse)	Day 67–70	Return within 24 h of discharge; hypotension; O_2_ saturation 70s overnight	300.8	5528	Interval worsening consolidation/ground-glass (Figure 1b)	200 mg SC twice daily restarted	High-dose IV methylprednisolone	Clinical improvement; anakinra Course 3 maintained

AFOP = acute fibrinous-organizing pneumonia; CT = computed tomography; DC = discontinuation; IV = intravenous; SC = subcutaneous; O_2_ = oxygen; PaO_2_/FiO_2_ = arterial oxygen tension/fraction of inspired oxygen.

## Data Availability

The original contributions presented in this study are included in the article. Further inquiries can be directed to the corresponding author.
